# Annual acknowledgement of manuscript reviewers

**DOI:** 10.1186/s12929-015-0122-6

**Published:** 2015-03-13

**Authors:** Wen-Chang Chang

**Affiliations:** Taipei Medical University, Taipei, Taiwan

## Abstract

The editors of *Journal of Biomedical Science* would like to thank all our reviewers who have contributed to the journal in Volume 21 (2014).

**Katsuyuki Aozasa**

Japan

**Bahram Arjmandi**

USA

**Li-Yuan Bai**

Taiwan

**Sarah Baird**

New Zealand

**Shahid Bashir**

USA

**Daniela Basso**

Italy

**Andrea Carmine Belin**

Sweden

**James Binley**

USA

**Claudia Biondi**

Argentina

**Ernst Bomhard**

Germany

**Renata Bortolus**

Italy

**Emanuela Bostjancic**

Slovenia

**Giacomina Brunetti**

Italy

**Chandana Buddhala**

USA

**Grant Campbell**

USA

**Duran Canatan**

Turkey

**Tai-Lung Cha**

Taiwan

**Siew Yeen Chai**

Australia

**Julie Chan**

Taiwan

**Samuel H.H. Chan**

Taiwan

**Alice Y.W. Chang**

Taiwan

**Long-Sen Chang**

Taiwan

**Ming-Fu Chang**

Taiwan

**Ming-Yang Chang**

Taiwan

**Su-Wei Chang**

Taiwan

**Tien-Hsien Chang**

Taiwan

**Tsuchung Chang**

Taiwan

**Yu-Ting Chang**

Taiwan

**Zee-Fen Chang**

Taiwan

**Yee Chao**

Taiwan

**Lee-Young Chau**

Taiwan

**Ben-Kuen Chen**

Taiwan

**Chieh-Fu Chen**

Taiwan

**Chien-Chang Chen**

Taiwan

**Dung-Tsa Chen**

USA

**Jaw-Wen Chen**

Taiwan

**Jen-Yang Chen**

Taiwan

**Jin-Chung Chen**

Taiwan

**Peilin Chen**

Taiwan

**Ruey-Hwa Chen**

Taiwan

**Ya-Wen Chen**

Taiwan

**Yen-Chou Chen**

Taiwan

**Yi-Jen Chen**

Taiwan

**Yi-Rong Chen**

Taiwan

**Yi-Ywan Margaret Chen**

Taiwan

**Jen-Kun Cheng**

Taiwan

**Yijuang Chern**

Taiwan

**Ya-Hui Chi**

Taiwan

**Jean-San Chia**

Taiwan

**Lih-Chu Chiou**

Taiwan

**Cheng-Hsun Chiu**

Taiwan

**Ing-Ming Chiu**

Taiwan

**Avijeet Chopra**

USA

**Chen-Kung Chou**

Taiwan

**Tz-Chong Chou**

Taiwan

**Yu-Ting Chou**

Taiwan

**Yen-Hung Chow**

Taiwan

**Lee-Ming Chuang**

Taiwan

**Shuang-En Chuang**

Taiwan

**Wan-Long Chuang**

Taiwan

**Chih-Pin Chuu**

Taiwan

**Marco Ciotti**

Italy

**Cristina Clement**

USA

**Dawn Coletta**

USA

**Luigi Del Vecchio**

Italy

**Shraddha Desai**

USA

**Santosh Dhule**

USA

**Hans Doerr**

Germany

**Roisin Dwyer**

Ireland

**Venessa Eeckhaut**

Belgium

**Bo Guang Fan**

Canada

**Fang Fang**

USA

**Kang Fang**

Taiwan

**Cathy SJ Fann**

Taiwan

**Kleber Farias**

Brazil

**Jie Feng**

USA

**David Fraser**

Australia

**Marcus Fruttiger**

UK

**Shu-Ling Fu**

Taiwan

**Wen-Mei Fu**

Taiwan

**Yasuhisa Fukui**

UK

**Padmaja Gade**

USA

**Kenneth Gagnon**

Canada

**Gabriel Galea**

UK

**Tom Gardiner**

UK

**SJ George**

UK

**David Gewirtz**

USA

**Stephen Ginsberg**

USA

**Goezde Girgin**

Turkey

**Gustavo Gonzales**

Peru

**Yuchun Gu**

China

**Jih-Hwa Guh**

Taiwan

**Natalia Gulyaeva**

Russia

**Yujie Guo**

USA

**Yutao Guo**

China

**Åsa Håkansson**

Sweden

**Peidong Han**

USA

**Xiaobin Han**

USA

**Robert Harris**

USA

**John Harwood**

UK

**Youssef Hatem**

Egypt

**Kiyoshi Hirahara**

Japan

**Yoshitaka Hirooka**

Japan

**Takayuki Hishiki**

Japan

**Siegfried Hoefinger**

Austria

**Guylaine Hoffner**

France

**Yoshiyuki Horio**

Japan

**Jim-Tong Horng**

Taiwan

**George Hsiao**

Taiwan

**Hsing-Pang Hsieh**

Taiwan

**Patrick Hsieh**

Taiwan

**Po-Shiuan Hsieh**

Taiwan

**Sung-Tsang Hsieh**

Taiwan

**Tao-Shih Hsieh**

Taiwan

**Chin Hsu**

Taiwan

**Hsin-Ling Hsu**

Taiwan

**Kuei-Sen Hsu**

Taiwan

**Ching-Yu Huang**

Taiwan

**Eagle Yi-Kung Huang**

Taiwan

**Kun-Lun Huang**

Taiwan

**Li-Rung Huang**

Taiwan

**Shiang-Suo Huang**

Taiwan

**Shih-Ming Huang**

Taiwan

**Tur-Fu Huang**

Taiwan

**Xupei Huang**

USA

**Yi-Tsau Huang**

Taiwan

**Chien-Fu Hung**

USA

**Ivan FN Hung**

Hong Kong

**Jan-Jong Hung**

Taiwan

**Li-Man Hung**

Taiwan

**Wen-Chun Hung**

Taiwan

**Shiaw-Min Hwang**

Taiwan

**Bartholomew Ibeh**

Nigeria

**Aaron James**

USA

**Jae-Young Je**

Korea, South

**Meei Jyh Jiang**

Taiwan

**Shibo Jiang**

China

**Zheng Jiang**

USA

**MCecilia Johnson**

Chile

**Yuh-Shan Jou**

Taiwan

**Jean-Sebastien Joyal**

Canada

**Chi-Chang Juan**

Taiwan

**Hsueh-Fen Juan**

Taiwan

**Jyh-Lyh (Jerry) Juang**

Taiwan

**Hyunmin Jung**

USA

**Suh-Hang Juo**

Taiwan

**Shivali Justa**

USA

**Shinji Kamada**

Japan

**Hong-Yo Kang**

Taiwan

**Reiji Kannagi**

Taiwan

**Parimal Kar**

USA

**Murat Kasap**

Turkey

**Abba Kastin**

USA

**Luqman Khan**

India

**Ushio Kikkawa**

Japan

**Nam Deuk Kim**

Korea, South

**Subodh Kishore**

India

**Konrad Klinghammer**

Germany

**Sivaramakrishna Koganti**

USA

**Naoki Koide**

Japan

**Jan Korbel**

Germany

**Theodora Koromila**

USA

**Ralf Krahe**

USA

**John Kung**

Taiwan

**Cheng-Chin Kuo**

Taiwan

**Ching-Chuan Kuo**

Taiwan

**Wu-Hsien Kuo**

Taiwan

**Yu-Min Kuo**

Taiwan

**Sandro La Vignera**

Italy

**Ming-Derg Lai**

Taiwan

**Ming-Zong Lai**

Taiwan

**Kuo-Chung Lan**

Taiwan

**Ping-Yee Law**

USA

**Chau-Hwang Lee**

Taiwan

**Hsiao-Hui Lee**

Taiwan

**Hsiao-Yuan Lee**

Taiwan

**Huei Lee**

Taiwan

**Ming-Shyue Lee**

Taiwan

**Oscar Lee**

Taiwan

**T-C Lee**

Taiwan

**Tzong-Shyuan Lee**

Taiwan

**Yi-Hsuan Lee**

Taiwan

**Steve Leu**

Taiwan

**Nicolas Leveque**

France

**Chao Li**

USA

**Chuan-Yuan Li**

USA

**Ling-Hui Li**

Taiwan

**Wan-Chun Li**

Taiwan

**Fang Liao**

Taiwan

**Yung-Feng Liao**

Taiwan

**Yen-Chywan Liaw**

Taiwan

**Chao-Hsiung Lin**

Taiwan

**Chih-Lin Lin**

Taiwan

**Chin-Tarng Lin**

Taiwan

**Chiou-Feng Lin**

Taiwan

**Chun-Yen Lin**

Taiwan

**Dingbo Lin**

USA

**Hsun-Hsun Lin**

Taiwan

**Jen-Der Lin**

Taiwan

**Kwang-Huei Lin**

Taiwan

**ShuWha Lin**

Taiwan

**Shu-Yi Lin**

Taiwan

**Su-Fang Lin**

Taiwan

**Wan-Wan Lin**

Taiwan

**Wen-Chang Lin**

Taiwan

**Ya-Wen Lin**

Taiwan

**Yi-Ling Lin**

Taiwan

**Jun-Yang Liou**

Taiwan

**Jyh-Ming Liou**

Taiwan

**Yih-Cherng Liou**

Singapore

**Chun-Jen Liu**

Taiwan

**Fu-Tong Liu**

USA

**Leroy Liu**

Taiwan

**Ping-Yen Liu**

Taiwan

**Shih-Tung Liu**

Taiwan

**Shing-Hwa Liu**

Taiwan

**Xiaochuan Liu**

USA

**Shih-Yen Lo**

Taiwan

**Rim Louati**

Tunisia

**Bin Lu**

USA

**Jean Lu**

Taiwan

**Lianghao Lu**

USA

**Yongjie Ma**

USA

**Donna MacCallum**

UK

**Nikolaus Maier**

Germany

**Maria Antonietta Maioli**

Italy

**Judith Choi Wo Mak**

Hong Kong

**Varpu Marjomäki**

Finland

**Olga Martínez-Augustin**

Spain

**Isao Matsuura**

Taiwan

**Toshimi Michigami**

Japan

**Ming-Yuan Min**

Taiwan

**Mario Minale**

Italy

**Marek Mirowski**

Poland

**Sameer Mirza**

USA

**Yasuo Miura**

Japan

**Petr Mlejnek**

Czech Republic

**Jigar Modi**

USA

**Kin Mok**

UK

**Garry Myers**

Australia

**Noriyo Nagata**

Japan

**Masaomi Nangaku**

Japan

**Kaei Nasu**

Japan

**Melyssa Negri**

Brazil

**Naoyuki Nishiya**

Japan

**Christian Noble**

Singapore

**Kandai Nozu**

Japan

**Michael O'Reilly**

UK

**Horng-Yih Ou**

Taiwan

**Gregory Oxenkrug**

USA

**Chunliu Pan**

China

**Hongbo Pang**

USA

**Li-Heng Pao**

Taiwan

**Alexander Pasternak**

Netherlands

**Cheng-Yuan Peng**

Taiwan

**Kristina Penniston**

USA

**Giulio Piluso**

Italy

**Howard Prentice**

USA

**Xia Pu**

USA

**Patimaporn Pungchanchaikul**

Thailand

**Weimin Qiu**

Denmark

**Hazir Rahman**

Pakistan

**Ram Reifen**

Israel

**Fernando Reis**

Brazil

**Randall Roper**

USA

**Akihide Ryo**

Japan

**Avneesh Saini**

USA

**Akikazu Sakudo**

Japan

**Doblin Sandai**

Malaysia

**Chiranjeevi Sandi**

UK

**Patricia Santos**

Brazil

**Dipak Sapkota**

Norway

**Stephen Schaffer**

USA

**Christine Sers**

Germany

**Joseph Shapiro**

USA

**Chen-Yang Shen**

Taiwan

**Chia-Ning Shen**

Taiwan

**Joen-Rong Sheu**

Taiwan

**Kak-Shan Shia**

Taiwan

**Sheau-Yann Shieh**

Taiwan

**Hsiu-Ming Shih**

Taiwan

**James Shih**

China

**Shin-Ru Shih**

Taiwan

**Hao-Ai Shui**

Taiwan

**Kou-Gi Shyu**

Taiwan

**Song-Kun Shyue**

Taiwan

**Rajvir Singh**

USA

**Dario Siniscalco**

Italy

**Andrew Smith**

UK

**Jiasheng Song**

USA

**Anna-Lena Spetz**

Sweden

**Supriya Srivastava**

Singapore

**Esta Sterneck**

USA

**Jin-Yuan Su**

Taiwan

**Ming-Jai Su**

Taiwan

**Tung-Hung Su**

Taiwan

**Yu-Wen Su**

Taiwan

**Chien-Feng Sun**

Taiwan

**Sunny Sun**

Taiwan

**Venkatesh Sundararajan**

USA

**Man-Sun Sy**

USA

**Nelofer Syed**

UK

**Ming-Hong Tai**

Taiwan

**You-Lin Tain**

USA

**Masanori Takahashi**

Japan

**Ming-Jer Tang**

Taiwan

**MI-Hua Tao**

Taiwan

**Pao-Luh Tao**

Taiwan

**Rui Tao**

USA

**Woan-Yuh Tarn**

Taiwan

**Marco Tartaglia**

Italy

**Daniel Tartakovsky**

USA

**Gamil Tawadrous**

Egypt

**Marc Tennant**

Australia

**Chenxi Tian**

USA

**Sokol Todi**

USA

**Martin Tolstrup**

Denmark

**Kuo-Wang Tsai**

Taiwan

**Chia-Lan Tsai**

Taiwan

**Ching-Hwa Tsai**

Taiwan

**Harley Tse**

USA

**Chien-Te Kent Tseng**

USA

**Ching-Jiunn Tseng**

Taiwan

**Hsien-Chun Tseng**

Taiwan

**Tai-Chung Tseng**

Taiwan

**Maria Tsironi**

Greece

**Kai-Yuan Tzen**

Taiwan

**Shun-Fen Tzeng**

Taiwan

**Mitsuhiro Uchiba**

Japan

**Natsuo Ueda**

Japan

**Haluk Vahaboglu**

Turkey

**Ineke van der Burgt**

Netherlands

**Cees Vermeer**

Netherlands

**Feng-Sheng Wang**

Taiwan

**Hsei-Wei Wang**

Taiwan

**Jia-Yi Wang**

Taiwan

**Jin-Town Wang**

Taiwan

**Jiun-JR Wang**

Taiwan

**Jiu-Yao Wang**

Taiwan

**Ju-Ming Wang**

Taiwan

**Paulus S. Wang**

Taiwan

**Shao-Win Wang**

Taiwan

**Shyi-Wu Wang**

Taiwan

**Tongfei Wang**

USA

**Tsu-Wei Wang**

Taiwan

**Wen-Chen Wang**

Taiwan

**Ying Wang**

USA

**Yun Wang**

Taiwan

**Zhao Wang**

USA

**Jenny Wei**

USA

**Wei-Zen Wei**

USA

**Yau-Huei Wei**

Taiwan

**David Weliky**

USA

**Andrew Whatmore**

UK

**David Williams**

USA

**Monique Williams**

South Africa

**Martin Witzenrath**

Germany

**Chih-Shung Wong**

Taiwan

**Bin-Nan Wu**

Taiwan

**Chin-Chen Wu**

Taiwan

**Deng-Chyang Wu**

Taiwan

**Gwo-Jang Wu**

Taiwan

**Han-Chung Wu**

Taiwan

**Jaw-Ching Wu**

Taiwan

**Jer-Yuarn Wu**

Taiwan

**Jinhua Wu**

USA

**Kay LH Wu**

Taiwan

**Kou-Juey Wu**

Taiwan

**Ming-Shiang Wu**

Taiwan

**Qingyu Wu**

China

**Sheng-Nan Wu**

Taiwan

**Xiwen Xiong**

USA

**Yi Xu**

China

**Xiaoyu Xue**

USA

**Takashi Yamatodani**

Japan

**Seiya Yamayoshi**

Japan

**Chuen-Mao Yang**

Taiwan

**Hongbo Yang**

USA

**Rong-Sen Yang**

Taiwan

**Ruey-Bing Yang**

Taiwan

**Shun-Fa Yang**

Taiwan

**Wei-Shiung Yang**

Taiwan

**Mumtaz Yaseen**

USA

**Hung-I Yeh**

Taiwan

**Kun-Huei Yeh**

Taiwan

**B. Linju Yen**

Taiwan

**Hsiu-Chuan Yen**

Taiwan

**Jiin-Cherng Yen**

Taiwan

**Mao-Hsiung Yen**

Taiwan

**Ruoh-Fang Yen**

Taiwan

**Shaw-Fang Yet**

Taiwan

**Hon-Kan Yip**

Taiwan

**Wenzhen Yu**

China

**Chun-Keung Yu**

Taiwan

**Ming-Jiun Yu**

Taiwan

**Ming-Lung Yu**

Taiwan

**Ke Yuan**

USA

**Ti-Fei Yuan**

China

**Vladimir Zarubaev**

Russia

**Qi Zeng**

USA

**Yaxue Zeng**

USA

**Jiangwei Zhang**

USA

**Yong Zhang**

China

**Ming Zhao**

USA

**Mingxin Zuo**

USA

